# Mental health care providers’ perceptions of the barriers to suicide prevention amongst people with substance use disorders in South Africa: a qualitative study

**DOI:** 10.1186/s13033-017-0153-3

**Published:** 2017-08-11

**Authors:** Daniel Goldstone, Jason Bantjes

**Affiliations:** Department of Psychology, Stellenbosch University, Stellenbosch, South Africa

**Keywords:** South Africa, Suicide prevention, Substance use disorder, Mental health care, Qualitative research, Low- and middle-income country

## Abstract

**Background:**

Substance use is a well-established, and potentially modifiable, risk factor for suicide. Suicide prevention interventions are typically framed within the biomedical paradigm and focus on addressing individual risk factors, improving access to psychiatric care, and improving the skills of medical personnel to recognise at-risk individuals. Few studies have focused on contextual factors that hinder suicide prevention in people with substance use disorders, particularly in low-resource settings. The aim of this qualitative study was to explore mental health care providers’ perceptions of barriers to suicide prevention in people with substance use disorders in South Africa.

**Methods:**

Semi-structured interviews were conducted with 18 mental health care providers who worked with suicidal people with substance use disorders in Cape Town, South Africa. Data were analysed using thematic analysis and Atlas.ti software was used to code the data inductively.

**Results:**

Two superordinate themes were identified: structural issues in service provision and broad contextual issues that pose barriers to suicide prevention. Participants thought that inadequate resources and insufficient training hindered them from preventing suicide. Fragmented service provision was perceived to lead to patients not receiving the psychiatric, psychological, and social care that they needed. Contextual problems such as poverty and inequality, the breakdown of family, and stigma made participants think that preventing suicide in people with substance use disorders was almost impossible.

**Conclusions:**

These findings suggest that structural, social, and economic issues serve as barriers to suicide prevention. This challenges individual risk-factor models of suicide prevention and highlights the need to consider a broad range of contextual and socio-cultural factors when planning suicide prevention interventions. Findings suggest that the responsibility for suicide prevention may need to be distributed between multiple stakeholders, necessitating intersectoral collaboration, more integrated health services, cautious use of task shifting, and addressing contextual factors in order to effectively prevent suicide in people with substance use disorders.

## Background

Suicidal ideation and behaviour (SIB) is a major public health concern in South Africa (SA) [1], and it is estimated that over 100,000 suicide attempts will be made in SA in 2017 [2]. SA has high rates of substance use disorders (SUDs) [3, 4] and substance use is a well-established risk factor for fatal and non-fatal suicidal behaviour [5–7]. The fact that people with SUDs (PWSUDs) constitute a large and well-delineated group that is at high risk of suicide, suggests that they should be a population targeted for specific suicide prevention interventions. This makes it important to better understand the context in which SIB occurs in PWSUDs and the contextual factors that might hinder suicide prevention in this population of health care users. However, little is known about possible barriers to suicide prevention in PWSUDs, particularly in low-resource contexts like SA. The aim of this study was to explore mental health care providers’ (MHCPs) insights into preventing suicide in PWSUDs, with a focus on MHCPs’ perceptions of possible barriers to suicide prevention.

### Suicide and substance use disorders

SUDs are well-established risk factors for both fatal and nonfatal suicidal behaviour [5–7]. Substance use is an independent risk factor for suicidal ideation [8], which is itself a risk factor for suicide [9]. Studies show that past-month prevalence of suicide ideation, plan, and attempt among PWSUDs may be as high as 20, 10, and 30%, respectively [10–12]. PWSUDs who seek treatment are approximately 9.8 times more likely to die by suicide than the general population [13]. There is also evidence that individuals who are intoxicated at the time of presenting for treatment following an incident of suicidal behaviour are less likely to be admitted to hospital or to be seen by a psychiatrist because of problems with the stigma associated with SUDs [14, 15].

Studies investigating SUDs and SIB are relatively scarce in low- and middle-income countries (LMICs) [16], although evidence shows that SUDs are consistently associated with SIB in LMICs [6]. A review of studies focused on injecting drug use has shown that the prevalence of co-morbid psychiatric disorders in PWSUDs in LMICs is high compared to the general population and is comparable to high-income countries [16]. Other studies provide evidence that the lifetime prevalence of suicidal ideation in PWSUDs in LMICs may be as high as 93%, while the lifetime prevalence of suicide attempts may be as high as 87%, although figures range between 50–93 and 43–87%, respectively [17–19].

In SA, nationally representative data estimate a 2.9% lifetime prevalence of suicide attempts and a 9.1% lifetime prevalence of suicidal ideation in the general population [20]. Between 7 and 30% of South Africans are high risk/problematic drinkers [21, 22], with 13.3% of the population reporting a lifetime diagnosis of a SUD [3]—higher than in most European countries [23]. Additionally, high rates of alcohol use (37%), marijuana use (10%) and tobacco use (25%) significantly predict suicidal behaviour among SA youths [4]. Together, these studies highlight the importance of understanding factors influencing suicide prevention in PWSUDs.

### Health care provision in SA

Health care provision in SA has been shaped by the country’s political history. In the apartheid era, the tricameral arrangement of the government led to health service provision being separated by race (white, coloured and Indian), with health care provision for black populations being provided by homeland administrations [24]. This led to inequalities in the availability of resources and resultant differences in health outcomes for people belonging to different race groups. Other issues identified between the period of 1960–1994 were fragmentation of services, constraints in the provision of psychiatric services, shortages in staff, poor public education about health, and increased focus on the private sector [24]. The National Health Plan of 1986 sought to rectify many of these issues and established the current tiered model of health care provision, but continued to encourage privatisation of health care [25].

After the democratic election in 1994, many apartheid-informed health policies were abolished, but the race and social class inequities in access to and utilisation of health care remained. New health care policies were aimed towards unifying fragmented services, reducing disparities and inequities, and improving access to resources [26, 27]. Access to health care was viewed as a human right, and the state was tasked with providing health care for all citizens. Intersectoral collaboration between different government departments was outlined as important to improve health outcomes, and the focus of health policies was to attend specifically to the needs of the most vulnerable groups [28].

Currently, health care in SA is provided by two parallel systems (public and private health care). Public health care is provided in a tiered system [29] and services more than 80% of the SA population [30]. However, the system is inundated and faces multiple challenges such as a high burden of disease and a lack of staff, resources, and infrastructure [31, 32]. The release of the National Mental Health Policy Framework and Strategic Plan, 2013–2020 [33] indicated a shift towards provision of person-centred mental health care that is integrated into primary health care (PHC). However, the aforementioned issues in the health system have obstructed such integration at a PHC level [34].

Service provision for PWSUDs is divided between the Department of Health (DOH; responsible for the medical and mental health needs of PWSUDs) and the Department of Social Development (DSD; responsible for the prevention and treatment of SUDs). This organizational structure reflects more global trends to deal with SUDs and mental health separately (for example, in the World Health Organization). Given the high comorbidity between SUDs and other psychiatric disorders [35], it is unclear why these issues are separated or what impact this has on service provision and suicide prevention.

### Suicide prevention

Research shows that clinicians’ abilities to predict suicide based on the assessment of risk factors is no better than chance [36]. Despite this, current approaches to suicide prevention are typically premised on individual risk-factor models, with interventions typically being aimed at identifying at-risk individuals, putting them in contact with MHCPs, and attenuating risk factors [37]. Interventions such as means restriction, effective treatment of psychiatric disorders, and improving the skills of PHC workers and general practitioners to screen for suicide and depression have shown some success in reducing SIB [37–39]. However, there is little to no evidence that these interventions take into account contextual factors that may influence their effectiveness. Particularly in settings where resources are limited, there may be contextual factors (social, economic, and cultural) that make these approaches unsuitable. At least one study has shown that a lack of resources, training, and time are perceived as barriers to suicide prevention in low-resource settings [40]. Another study has shown that infrastructural and familial factors were associated with suicide attempts in PWSUDs, while more established risk factors such as depression and anxiety were not [41]. This highlights the importance of understanding the context of suicide, how different high-risk populations may experience specific risk factors, and how contextual factors within the health care system may hinder suicide prevention. It is unclear at present what contextual factors may act as barriers to suicide prevention in the context of providing care to PWSUDs in SA.

Recent research shows that patients who have attempted suicide in SA may not be receiving the psychological and psychosocial support that they require in emergency psychiatric units [42]. This is supported by other SA studies showing that psychiatric patients in general do not receive the psychosocial care that they need and that doctors and nurses in PHC facilities often feel insufficiently trained to deal with psychiatric emergencies [43, 44]. No research has been done in the SA context to investigate whether MHCPs perceive these or other issues to be important in the context of suicide prevention in PWSUDs.

## Methods

### Aim, design, and setting

The aim of this study was to explore MHCPs’ insights into preventing suicide in PWSUDs, with a focus on MHCPs’ perceptions of possible barriers to suicide prevention. The qualitative design of this study allowed for exploratory investigation of an under-researched topic of much clinical relevance to MHCPs, particularly those working in LMICs. The study setting was the South African health care system, which has been described as overburdened and understaffed, as it faces a high burden of disease and a relative lack of resources [31, 32].

### Sampling and participants

Purposive and snowball sampling were utilized to recruit participants. JB has worked in mental health in SA for over a decade and is well acquainted with both public and private health care settings. JB was able to suggest potential participants who worked in mental health, had experience working with suicidal PWSUDs, and would be able to provide insight on the topic. These potential participants were invited to participate (the purposive phase of the sampling procedure) and were asked if they could recommend colleagues who would be able to add value to this study (the snowball phase of the sampling procedure). As a result, MHCPs from a range of professions (psychiatry, psychology, social work, and counselling) were invited to participate. Three potential participants did not respond to invitations to participate, 18 consented, and none refused to participate. Collecting data from MHCPs in different professions allowed us to capture the same issue from multiple perspectives, enhancing methodological triangulation.

### Data collection

In-depth, semi-structured interviews were conducted in English with 18 MHCPs by DG. Each interview lasted between 40 and 90 min and was conducted at a time and place of the participant’s choice. Participants were asked about their experiences with preventing suicide in PWSUDs, and were asked to focus particularly on what they perceived to be barriers to suicide prevention in PWSUDs. All interviews were digitally audio-recorded and transcribed. Data were collected between 02 September and 30 November 2016.

### Data analysis

The data were analysed using thematic analysis [45]. Thematic analysis allows for codes and themes to be generated inductively through reflective engagement with the interview transcripts [45]. Codes are considered the smallest meaning units in the data and represent elements of the raw data that appear interesting or appear to be relevant to the research question(s) [45, 46]. Generating codes inductively (using a data-driven approach) entails scrutinizing the transcribed text to identify what the meaning behind the participant’s communication might be, and then assigning a code to each section of text (be it a word, phrase, or paragraph). To do this, DG read the data multiple times to familiarise himself with them, and then coded the data inductively. JB reviewed the codes by cross-checking them against the interview transcripts. This was done to ensure that there was agreement about each code’s meaning, to check whether there were other possible interpretations of the data, and to ensure that each code made sense. This led to reassignment of some codes.

After the coding process, both authors grouped the codes into themes that captured the underlying data. The authors did this by meeting weekly during the analysis process to discuss the codes and themes, to ensure that all aspects of the data were being captured and that the themes and their meanings were mutually agreed-upon. Themes are broader meaning units that show a greater and more integrated understanding of the meaning of the data [45]. Themes were generated by grouping similar codes together, identifying what their underlying semantic and latent similarities were, and assigning a name to the theme that best represented the meaning of the underlying codes and data.

The authors took a number of measures to enhance the trustworthiness of the data. Ensuring trustworthiness in qualitative research is a way to provide evidence for the methodological rigour of the study, comparable to ensuring reliability and validity in quantitative research [47, 48]. Trustworthiness can be enhanced in terms of four criteria: credibility, transferability, dependability, and confirmability [47].

Credibility refers to the amount of agreement between the data and the perceived reality of the participants [47, 48]. We tested the credibility of our findings using on the spot member checking, which entailed asking participants clarifying questions during the interviews, to ensure that our understanding of what the participants were saying matched theirs [49]. Quotes are reported in the findings to provide evidence for the themes and enhance the credibility of the findings.

Transferability refers to the extent to which the findings of a study might be applicable in similar contexts [47]. It is possible that if there are inherent similarities between study participants, contexts and research questions, similar results will be found. By using purposive sampling, providing a thick description of the findings [50], providing sufficient data, and contextualising the findings of this study in the discussion, we enhance the ability of readers to assess the transferability of these results to similar contexts with which they are familiar [47, 51, 52].

Dependability refers to whether the variance in the method of data collection and analysis can be tracked as the research process unfolds, so that changes in reality or in the meaning of the data and the reasons behind these changes can be identified [47]. To ensure dependability, the data collection and analysis process was recorded in detail using comprehensive field notes and reflections on the research process.

Finally, the findings of a study are confirmable if the data collection and analysis process has been sufficiently detailed in the written report, the limitations of the study have been noted, and evidence of the researchers’ identifications of their own biases has been provided [47]. An important part of confirmability is bracketing, which refers to recognising and setting aside one’s own knowledge, preconceptions, biases, opinions, and a priori assumptions about the research topic with the intent of being open minded and maximally receptive to participant experiences [53, 54]. The authors reflected on their knowledge about the SA health care system and made a conscious effort to set aside any personal biases about what might be found, to allow the participants’ accounts of their perceptions to unfold as organically as possible.

Data from all 18 interviews are included in the findings, to ensure that there was no bias towards or against any participants. The interview transcripts were analysed using the software program Atlas.ti. The data presented here form a subset of the total findings. This article focuses on themes relating to barriers to suicide prevention. The remainder of the findings deal with MHCPs’ experiences of preventing suicide in PWSUDs and what their suggestions are for suicide prevention in PWSUDs. Those findings will be reported elsewhere.

## Results

Two superordinate themes were identified in the data: structural issues in service provision and contextual issues that extend beyond health care. Structural issues such as (a) a lack of resources, (b) insufficiencies in training, and (c) fragmentations in the organisation of care led participants to think that many suicidal PWSUDs do not receive the psychiatric, psychological, and social care that they need. Participants thought that contextual issues, namely (a) poverty and inequality, (b) the breakdown of family, and (c) stigma made it difficult to effectively prevent suicide in PWSUDs. Together, these factors acted as barriers to suicide prevention and led participants to feel hopeless and powerless in their work preventing suicide in PWSUDs.

### Structural issues in service provision

#### A lack of resources

Participants said suicide prevention was hampered by a widespread lack of resources in an overburdened system. Insufficient emergency psychiatric services and the thin spread of specialised MHCPs across health facilities meant that “*service provision is inundated and clogged up by seriously ill patients,*” who were then prioritised ahead of those with problems that were perceived as less serious. This led many to think that mental health, especially substance use and SIB, is relegated to the bottom of the government’s list of health care priorities. The lack of resources, especially the lack of specialised services for SIB and SUDs, often prevented patients from receiving the care that they needed, with Shaun expressing that “*we had four suicides in one year… [and] I believe they were all let down by the system as a whole.*”

Many participants said they were frustrated because they were unable to implement best practices due to severe time and funding constraints. Participants working in SUD treatment facilities financially supported by the government thought that the lack of available funding was responsible for needing to follow a “*standardized treatment plan*” in order to meet “*targets*”. This was perceived to limit the quality and duration of care that MHCPs are able to provide, with Tatum expressing that “*sometimes it’s more important just to focus on the relationship with the client… because I think at the end of the day, most of our clients already know most of what we’re going to tell them.*” Having limited time to provide services meant that participants often had to refer patients who needed services to other overburdened facilities. Frank explicitly expressed his own distrust in the system, stating that “*[the health care system is] very bleak, I fear for if I feel suicidal one day. I don’t trust the system because I don’t know how well the system is functioning.*” Similarly, Sophie expressed that the lack of a holistic treatment approach and the lack of social services made her job as a psychologist more difficult:

*Just in terms of social things like living, where can people live, where can they stay? Also simple things, like assisting people with getting ID documents, assisting people with places where they can wash, there’s all sorts of things, and I think that’s really neglected, I mean, again someone with a substance use disorder, where do they go?*

#### Insufficiencies in training

Participants with only four years of training (counsellors and social workers) explained how their university education had not prepared them to adequately screen for, identify, and manage SIB. Many noted that they thought they needed continuous post-graduate training to “*keep on top of new literature*” and best practices in order to prevent suicide effectively.

The insufficiencies in training were perceived to be more severe for health personnel who were not MHCPs. Emergency services and staff at day hospitals are usually the first people to come into contact with suicidal PWSUDs, but participants thought that they often mismanaged these patients. This made participants hesitant about referring suicidal PWSUDs to health facilities that were supposed to provide services for suicidal PWSUDs. Despite some of their own perceived gaps in training, they said that they were doing their best to prevent suicide but thought that it was made more difficult when medical personnel did not take suicide risks seriously.

#### Fragmentations in the organisation of care

Multiple participants used the phrase “*falling through the cracks*” to describe how suicidal PWSUDs often did not receive the mental health care that they required, which was thought to result from the way that health care in SA is organised. Participants said that the tiered system of service provision and standard process of referral from specialist SUD treatment facilities, to primary health care facilities, to secondary or tertiary facilities caused major delays before suicidal PWSUDs were able to receive admission for suicidality. Participants related experiences of referring high-risk suicidal patients to a hospital for admission, and then having patients be turned away. Discharge of patients who were imminent suicide risks from health care facilities led participants to feel despondent about suicide prevention.

Part of the fragmentation in health care provision was thought to result from the split between public and private health facilities. One participant highlighted the blatant inequality between private and public health care settings, stating that “*if you don’t have a medical aid, and you’ve got a substance use problem in this country, you are in a very, very difficult situation.*” The lack of SUD treatment facilities in the public sector means that patients must “*wait like two or three months to see the substance use doctors*.” Conversely, in the private sector patients have to pay high fees to MHCPs. Even for those who can afford private care, many are limited by their medical aid policies and the restrictions placed on what types of care are covered. Participants said that both scenarios made patients feel unwelcome and uncared for, giving patients the perception that the health care system (public or private) does not have their best interests at heart. This led participants to conclude that PWSUDs are left feeling “*complete and utter frustration with [not feeling like your problems are important] and an inability to actually access good quality care, acutely, and also afterwards as an out*-*patient [that leads to PWSUDs becoming suicidal].*”

Participants working at substance use rehabilitation centres often distinguished SUDs from mental health issues, saying that mental health issues must be resolved and a person must be non-suicidal before they could address the person’s SUD. Conversely, psychiatric/medical staff thought that a patient’s SUD should be addressed before the mental health issues could be treated. Conceptualising SUDs and mental health as separate issues reflected the “*very weird split*” between the DOH and DSD. This splitting in service provision meant that “*substance use is the portfolio of the Department of Social Development [but] the Department of Social Development doesn’t provide the health services that are needed.”* Participants thought this reflected “*no real coherence [as] everyone’s just sort of doing their own thing, and there’s just this sort of turn over, but it’s not really addressing the underlying problem, or the cause.*” Poor follow-up systems and a lack of communication between MHCPs made it “*very difficult*” for participants as they didn’t know what was happening to patients who were suicidal and had been discharged from health or SUD treatment facilities or referred to others. Many said that this made it difficult to know whether they were preventing suicides at all.

### Contextual issues extending beyond health care

#### Poverty and inequality

Some participants outlined how the vast inequality and poverty in SA are economic and social after-effects of apartheid and are clearly evident in the lives of PWSUDs. This tied into high levels of trauma and violence that PWSUDs experienced, and participants theorised that this played an undeniable role in the substance use and SIB of their patients. Josephine told of one patient who had ended up in prison:

*The reason why she ended up in prison was because she had tried to take her own life and her child’s life. And she’d done that twice…. Each time she was completely drunk, but when I examined her life… there was a lot of rape, a lot of physical abuse from very young and also a lack of complete hope that things would change for her child. So I said to her, “But why would you take the child’s life?” You know? And she said, “Because I could see her life going the same way as mine and I was afraid for her, and I couldn’t leave her, because then she would be without anybody, and so I thought I’d take both of our lives.”*

Many participants perceived SUDs to be *“a symptom of what’s happening in our communities*” and *“a social disease that actually results in medical changes in the brain”* as a result of *“unemployment, disenfranchisement, lack of representation of local government, gangsterism, domestic violence, disintegrated social fabric, [and] substance abuse in families.*” Participants empathised with their clients and thought it was understandable that someone would feel suicidal if they experienced the problems listed above. Participants said that for many PWSUDs, there is no meaningful alternative to substance use. As a result, participants thought that removing their “*coping mechanism*” has detrimental effects on their psychological wellbeing and often causes SIB.

The poverty that suicidal PWSUDs experienced became a very real factor in the therapeutic environment when participants tried to help patients restructure their lives. Participants told of needing to go above and beyond their responsibilities or scope of practice as MHCPs to help their patients. Providing patients with “*bus fare*”, “*helping a lot of them with drawing up CVs*”, or walking them to a hospital “*wasn’t my job*” but became part of preventing suicide, because patients were so poor or poorly educated that they could not do these things themselves. Poverty not only created the conditions under which people felt suicidal, but was also a major barrier to addressing this suicidality.

#### The breakdown of family

As with poverty and inequality, participants said that the “*breakdown… in family in the society that we live in*” reflected a much broader societal issue that served as a barrier to suicide prevention in PWSUDs. Participants said that family often played a central role in the lives of PWSUDs, either in directly causing the person’s substance use, or in contributing to the continuation of the person’s use and their SIB. Having poor role models and parents who used substances was thought to be a major factor causing some patients’ substance use and SIB. In other cases, many PWSUDs “*are highly displaced people who have been kicked out by their families… either the drug use was an excuse, or they were excluded prior to their drug use becoming a big problem.*” Participants said that even when a PWSUD exists within a family system, they are often deeply rejected and receive no support, love and care from that system. As a result, treating someone for SIB and then sending them back into the very environment that made them suicidal was thought to be an ineffective way to manage SIB, and is a poignant reflection that suicide, like substance use, is a community and social problem and not an individual one.

#### Stigma

Stigma against suicidal PWSUDs was thought to be a major barrier to suicide prevention, for two reasons. First, suicide is “*still so stigmatised, that it’s very difficult for people to access the help that they really need without being vilified and stigmatised.*” This “*help*” referred to both formal mental health services and informal support from family and friends. PWSUDs were treated “*almost like they’re not a human, like they’re not a person, they’re just a drug addict.”* The continued presence of stigma and dehumanisation was believed to result in PWSUDs identifying with the stigma and stigmatising themselves, meaning that “*[not] all substance users present for treatment.*” Stigma was perceived to be such a powerful barrier to suicide prevention in PWSUDs that Insaaf said “*I don’t know what can be done with the suicide thing besides the stigma. Because just going for help in general is like almost seen as, ‘You weak,’ or, ‘You mad,’ or, ‘You crazy’.*”

Second, stigma prevented suicidal PWSUDs from speaking about their suicidality even when they were receiving help. The stigma that came from medical professionals was especially condemning and was believed to traumatise patients deeply, with Berkeley noting that “*people think if [PWSUDs] do hurt themselves or they’re suicidal it’s kind of like… ‘Well it’s, they better off dead,’ this is what our colleagues [think], these are the kind of things you hear.”* This was another factor that made participants very reluctant to refer suicidal PWSUDs to health facilities. When PWSUDs concealed their suicidality, it was “*much more scary than the one that’s… actually telling you ‘I’m gonna go get my father’s gun and I’m gonna shoot myself,’* as participants could then “*respond to the emergency,”* while “*when things are hidden, that’s really scary.”*

Stigma was understood to be a result of many things. The lack of knowledge amongst the general population about how to deal with SIB and the fear that many feel about discussing SIB was thought to give rise to stigma. Similarly, the belief that mental illnesses (including SUDs and SIB) “*do not exist”* or are a moral failing in the person was also thought to be a reason that SIB and SUDs were stigmatised. JC attributed the rejection of suicidal PWSUDs and the shame and embarrassment that surrounds suicide and SUDs to the conservative mindset of many South Africans:

*South Africa, it’s a very conservative country… and a lot of that is probably because of the influence of you know religion, conservative upbringing, this idea that if you commit suicide, you’re gonna go to hell [or] bring shame upon the family [or] the life insurance policy may not pay out [or] “if my child commits suicide, it means that I’m a bad parent”… so society stigmatizes a condition because it doesn’t understand the condition.*

Participants said that the rejection that PWSUDs experienced from their families and communities led to deep feelings of shame and embarrassment about their substance use and SIB, which in turn made their substance use and SIB worse. A cycle developed of substance use leading to shame, embarrassment and SIB, with rejection and lack of support compounding these feelings, leading to further substance use, shame, embarrassment and SIB, and so on.

## Discussion

Participants in this study highlighted that a lack of training makes preventing suicide in PWSUDs difficult, especially because they perceived the SA health care system to be under-resourced and overburdened. Overburdened health systems and a lack of resources are common in LMICs [55] and have been identified as barriers to providing adequate care for suicidal patients and PWSUDs [42, 56]. Additionally, inadequate training and experience in suicide prevention diminishes the competencies of health care providers to respond appropriately to suicidal patients [57]. To reduce the burden on health systems, task-shifting is often utilised or proposed as a cost-effective method of transferring the care of patients to MHCPs with comparatively less training (such as counsellors and social workers) [58, 59]. However, the experiences of these participants question the usefulness of task-shifting when MCHPs are not prepared to manage suicidal patients. While more services are needed, current legislation governing who can provide services for PWSUDs in SA does not clearly articulate the minimum skills and competencies required by service providers [60, 61]. There is no indication that training to manage suicide crises is mandatory. Further training of MHCPs in targeted suicide prevention strategies may be required to strengthen current task-shifting models of care. Training medical personnel to be more empathic with suicidal individuals and to accurately assess suicide risk may also be an important way to ensure that fewer patients are turned away when seeking care.

Many of the fragmentations and socioeconomic issues identified by the participants in this study are historical artefacts from apartheid-era SA. While significant steps have been taken to rectify these issues, the segregation between public and private health care continues to underserve patients and undermine suicide prevention in PWSUDs. With a current unemployment rate of 27.1% and more than half the population living below the national poverty line [62, 63] it is clear that population-wide poverty and inequality that resulted from apartheid policies have still not been addressed. Poverty and inequality are established risk factors for SIB [64] and SUDs [65], and the combination of poverty and substance use is a strong predictor of first-time suicide attempts [66]. With one SA study showing that 56.9% of individuals who died by suicide over a 5 year period were unemployed [67], it is evident that poverty and inequality are relevant risk factors for suicide in SA. Taken together, this shows that contextual factors may be as important as individual risk factors for suicide prevention in PWSUDs.

Participants in this study say that they cannot take sole responsibility for suicide prevention because they believe there are social, economic and cultural factors that give rise to the circumstances under which people develop SUDs and under which PWSUDs become suicidal. The split between the DSD and DOH was believed to add to these issues by creating diffusion of responsibility regarding who should provide care for these patients. This highlights the apparent difficulty of being a health care provider tasked with preventing suicide when there are much broader factors at play influencing suicide prevention. This brings into question the scope of the role of the health care provider. On one hand, health care providers have a medical and legal responsibility to prevent suicide, but on the other they cannot be expected to be solely responsible for suicide prevention given the perceived social, economic, and cultural barriers to suicide prevention. It may be important in this regard to open up healthier and more collaborative conversations about suicide between MHCPs and other stakeholders involved in preventing suicide.

As such, more integration and intersectoral collaboration between different health care services, policy makers, and government departments appears to be required so that the responsibility for suicide prevention can be shared. Such integrated approaches have been proposed in both the National Drug Master Plan 2013–2017 [68] and the National Mental Health Policy Framework and Strategic Plan 2013–2020 [33], although evidence for this integration is absent. Research has identified a lack of communication between sectors, problems delineating roles, and perceptions of not being supported by other sectors as some of the reasons for this lack of integration and intersectoral collaboration [69]. Suggestions for improving intersectoral collaboration have been recognised more generally for mental health in SA [69] but suggestions specific to suicide prevention are currently lacking.

Preventing suicide requires a careful understanding of a very complex phenomenon, and we lack precise models to predict suicide based solely on individual risk factors [36]. By focusing only on mental health, or SUDs, or social disintegration, we miss how these factors interact with one another and we miss broader factors related to health care seeking and suicide prevention. For example, stigma is a known barrier to mental health care seeking [70, 71], and was identified in this study as an important barrier to suicide prevention. Additionally, the organisation of care within the SA health system was also identified as a major barrier to suicide prevention. While it may be a uniquely South African phenomenon that services are so segregated, arising from the divisions between (a) public and private health care and (b) the DOH and DSD, it is apparent that the structural and organisational components of health care systems need to be considered in addition to individual risk factors when designing suicide prevention interventions.

Research shows that social, economic, and cultural issues are significantly linked to SIB [64, 72]. For example, in PWSUDs in India, social and economic issues (housing insecurity and poor family relationships) were associated with suicide attempts while mental health problems (depression and anxiety) were not [41]. Along with the findings of this research, this shows that PWSUDs appear to experience specific social and economic risk factors for suicide that may not apply to other high-risk groups [41]. This provides good reason to challenge and transform individual risk-factor models of suicide prevention in PWSUDs and move towards more comprehensive, context-specific models of understanding suicide and its prevention [41, 73, 74].

Addressing the contextual factors influencing suicide may be particularly important in the context of substance use in LMICs. Researchers have argued for the need to consider the structural determinants of suicide in PWSUDs in other LMICs, and have suggested a number of important strategies to help prevent suicide in PWSUDs [75]. Raising awareness of the high risk of suicide in PWSUDs, developing culturally appropriate suicide prevention guidelines, upskilling health care workers to screen for and manage suicide risk, addressing the psychosocial drivers of SIB by tending to housing, vocational and family crises, and moving towards a social model of recovery are just some of these suggestions [75]. Such comprehensive, socially-focused approaches to suicide prevention in PWSUDs have yet to be trialled and tested.

In the SA context, perhaps all that is needed is a re-purposing and reorganisation of existing resources. This will not necessarily decrease the burden on health care and social work systems, but by streamlining and improving the efficiency of care, it is likely that patients will be more adequately attended to. In the long term, this may lead to decreases in SIB and a resultant decrease in the burden on the health care system. The data from this study strongly suggest that there is a need to address socioeconomic and family problems in addition to mental health problems and the sequelae of SUDs. This may necessitate a more integrated model of care that extends beyond medicine and mental health to include a focus on social services, family support, and psychoeducation for the community at large.

## Limitations

The focus on the perspectives of MHCPs does not allow us to know what the opinions of suicidal PWSUDs are, or whether they perceive the same problems to be important in preventing SIB. The qualitative design of this research means that the findings cannot be reliably generalised to settings in other countries or possibly even other parts of SA. The findings indicate that preventing suicide requires a concerted multi-level effort from many stakeholders and may require broad-scale changes in society, which can only be effected over a long time and may not be feasible or realistic given limitations to government funding. Nonetheless, this study provides a useful first step in describing the barriers to suicide prevention perceived by MHCPs tasked with the responsibility of providing care for suicidal PWSUDs.

## Conclusions

The structural and contextual barriers to suicide prevention in SA identified in this study draw attention to the possible limitations of suicide prevention interventions premised on individual risk-factor models. Contextual issues need to be targeted and addressed as part of integrated suicide prevention strategies, particularly for high-risk populations like PWSUDs. In resource-limited settings, training MHCPs adequately in targeted suicide prevention interventions may be important for the success of task-shifting models of health care provision. Additionally, training medical personnel with better skills to accurately assess suicide risk and express more empathy with suicidal patients may help improve service provision and suicide prevention efforts. The current fragmented organisation and provision of services points to a need for more integrated services and intersectoral collaboration. This is not unique to suicide prevention as it is required to improve mental health care provision more generally. Finally, addressing fragmentations through intersectoral collaboration and increased integration may help distribute the responsibility for suicide prevention between various stakeholders, including government departments, MHCPs, families and communities, so that MHCPs feel supported and more able to prevent suicide.

## Abbreviations

DOH: Department of Health
DSD: Department of Social Development
LMICs: low- and middle-income countries
MHCP: mental health care provider
PHC: primary health care
PWSUD: person with a substance use disorder
SA: South Africa
SIB: suicidal ideation and behaviour
SUD: substance use disorder

## References

[1] Bantjes J, Kagee A (2013). Epidemiology of suicide in South Africa: setting an agenda for future research. S Afr J Psychol.

[2] Goldstone D. Suicide prevention in South Africa: research, praxis and policy. Manuscript under review.

[3] Herman AA, Stein DJ, Seedat S, Heeringa SG, Moomal H, Williams DR (2009). The South African Stress and Health (SASH) study: 12-month and lifetime prevalence of common mental disorders. S Afr Med J.

[4] Shilubane HN, Ruiter RA, van den Borne B, Sewpaul R, James S, Reddy PS (2013). Suicide and related health risk behaviours among school learners in South Africa: results from the 2002 and 2008 National Youth Risk Behaviour Surveys. BMC Public Health.

[5] Borges G, Loera CR (2010). Alcohol and drug use in suicidal behaviour. Current Opin Psychiatry.

[6] Breet E, Goldstone D, Bantjes J. Substance use and suicidal behaviour in low- and middle-income countries: a systematic review. Manuscript under review. 10.1186/s12889-018-5425-6 PMC5921303 29699529

[7] Kennedy MC, Marshall BD, Hayashi K, Nguyen P, Wood E, Kerr T (2015). Heavy alcohol use and suicidal behavior among people who use illicit drugs: a cohort study. Drug Alcohol Depend.

[8] Pages KP, Russo JE, Roy-Byrne PP, Ries RK, Cowley DS (1997). Determinants of suicidal ideation: the role of substance use disorders. J Clin Psychiatry.

[9] Kessler RC, Borges G, Walters EE (1999). Prevalence of and risk factors for lifetime suicide attempts in the National Comorbidity Survey. Arch Gen Psychiatry.

[10] Manning V, Koh PK, Yang Y, Ng A, Guo S, Kandasami G, Wong KE (2015). Suicidal ideation and lifetime attempts in substance and gambling disorders. Psychiatry Res.

[11] Chen VC-H, Lin T-Y, Lee CT-C, Lai T-J, Chen H, Ferri CP (2010). Suicide attempts prior to starting methadone maintenance treatment in Taiwan. Drug Alcohol Depend.

[12] Lee TS-H, Shen H-C, Wu W-H, Huang C-W, Yen M-Y, Wang B-E (2011). Clinical characteristics and risk behavior as a function of HIV status among heroin users enrolled in methadone treatment in northern Taiwan. Subst Abuse Treat Prev Policy.

[13] Wilcox HC, Conner KR, Caine ED (2004). Association of alcohol and drug use disorders and completed suicide: an empirical review of cohort studies. Drug Alcohol Depend.

[14] Li YM (2007). Deliberate self-harm and relationship to alcohol use at an emergency department in eastern Taiwan. Kaohsiung J Med Sci.

[15] Ries RK, Yuodelis-Flores C, Comtois KA, Roy-Byrne PP, Russo JE (2008). Substance-induced suicidal admissions to an acute psychiatric service: characteristics and outcomes. J Subst Abuse Treat.

[16] Iskandar S, Kamal R, De Jong CA (2012). Psychiatric comorbidity in injecting drug users in Asia and Africa. Curr Opin Psychiatry.

[17] Armstrong G, Nuken A, Samson L, Singh S, Jorm AF, Kermode M (2013). Quality of life, depression, anxiety and suicidal ideation among men who inject drugs in Delhi, India. BMC Psychiatry.

[18] Erfan S, Hashim AH, Shaheen M, Sabry N (2010). Effect of comorbid depression on substance use disorders. Subst Abus.

[19] Sarin E, Samson L, Sweat M, Beyrer C (2011). Human rights abuses and suicidal ideation among male injecting drug users in Delhi, India. Int J Drug Policy.

[20] Joe S, Stein DJ, Seedat S, Herman A, Williams DR (2008). Prevalence and correlates of non-fatal suicidal behaviour among South Africans. Br J Psychiatry.

[21] Department of Health, Medical Research Council, OrcMacro (2007). South Africa demographic and health survey 2003.

[22] Parry CD, Plüddemann A, Steyn K, Bradshaw D, Norman R, Laubscher R (2005). Alcohol use in South Africa: findings from the first Demographic and Health Survey (1998). J Stud Alcohol Drugs.

[23] Kessler RC, Angermeyer M, Anthony JC, De Graaf RO, Demyttenaere K, Gasquet I (2007). Lifetime prevalence and age-of-onset distributions of mental disorders in the World Health Organization’s World Mental Health Survey Initiative. World Psychiatry.

[24] Van Rensburg HJC, Van Rensburg HJC (2012). A history of health and health care in South Africa 1652–1994. Health and health care in South Africa.

[25] Department of National Health and Population Development (1986). ‘n Nuwe bedeling: ‘n nasionale gesondheidsplan vir Suid Afrika.

[26] Department of Health (1996). Restructuring the national health system for universal primary health care.

[27] Department of Health (1997). White paper for the transformation of the health system in South Africa—Government gazette 17910 Notice 667 of 1997.

[28] Van Rensburg HJC, Engelbrecht MC, Van Rensburg HJC (2012). Transformation of the South African health system: post 1994. Health and health care in South Africa.

[29] Department of Health (2012). National Health Act, 2003: Regulations relating to categories of hospitals (Government Gazette, 02 March 2012, No. R. 185).

[30] Benatar SR (2013). The challenges of health disparities in South Africa. S Afr Med J.

[31] Coovadia H, Jewkes R, Barron P, Sanders D, McIntyre D (2009). The health and health system of South Africa: historical roots of current public health challenges. Lancet.

[32] Chopra M, Lawn JE, Sanders D, Barron P, Karim SS, Bradshaw D (2009). Achieving the health Millennium Development Goals for South Africa: challenges and priorities. Lancet.

[33] Department of Health (2013). National mental health policy framework and strategic plan 2013–2020.

[34] Marais DL, Petersen I (2015). Health system governance to support integrated mental health care in South Africa: challenges and opportunities. Int J Ment Health Syst.

[35] Kandel DB, Huang FY, Davies M (2001). Comorbidity between patterns of substance use dependence and psychiatric syndromes. Drug Alcohol Depend.

[36] Franklin JC, Ribeiro JD, Fox KR, Bentley KH, Kleiman EM, Huang X (2017). Risk factors for suicidal thoughts and behaviors: a meta-analysis of 50 years of research. Psychol Bull.

[37] Mann JJ, Apter A, Bertolote J, Beautrais A, Currier D, Haas A (2005). Suicide prevention strategies: a systematic review. JAMA.

[38] van der Feltz-Cornelis CM, Sarchiapone M, Postuvan V, Volker D, Roskar S, Grum AT (2011). Best practice elements of multilevel suicide prevention strategies: a review of systematic reviews. Crisis.

[39] Rutz W, Walinder J, von Knorring L, Rihmer Z, Pihlgren H (1997). Prevention of depression and suicide by education and medication: impact on male suicidality. An update from the Gotland study. Int J Psychiatry Clin Pract.

[40] Medina CO, Kullgren G, Dahlblom K (2014). A qualitative study on primary health care professionals’ perceptions of mental health, suicidal problems and help-seeking among young people in Nicaragua. BMC Fam Pract.

[41] Armstrong G, Jorm AF, Samson L, Joubert L, Singh S, Kermode M (2014). Suicidal ideation and attempts among men who inject drugs in Delhi, India: psychological and social risk factors. Soc Psychiatry Psychiatr Epidemiol.

[42] Bantjes J, Nel A, Louw K-A, Frenkel L, Benjamin E, Lewis I (2016). “This place is making me more depressed”: the organisation of care for suicide attempters in a South African hospital. J Health Psychol.

[43] Joska JA, Flisher AJ (2007). Needs and services at an in-patient psychotherapy unit. Afr J Psychiatry.

[44] Petersen I, Bhana A, Campbell-Hall V, Mjadu S, Lund C, Kleintjies S (2009). Planning for district mental health services in South Africa: a situational analysis of a rural district site. Health Policy Plan.

[45] Braun V, Clarke V (2006). Using thematic analysis in psychology. Qual Res Psychol.

[46] Boyatzis RE (1998). Transforming qualitative information: thematic analysis and code development.

[47] Guba EG (1981). Criteria for assessing the trustworthiness of naturalistic inquiries. Educ Technol Res Dev.

[48] Graneheim UH, Lundman B (2004). Qualitative content analysis in nursing research: concepts, procedures and measures to achieve trustworthiness. Nurse Educ Today.

[49] Mays N, Pope C (2000). Assessing quality in qualitative research. BMJ.

[50] Geertz C (1973). The interpretation of culture.

[51] Bradley J (1993). Methodological issues and practices in qualitative research. Libr Q.

[52] Shenton AK (2004). Strategies for ensuring trustworthiness in qualitative research projects. Educ Inform.

[53] Gearing RE (2004). Bracketing in research: a typology. Qual Health Res.

[54] van Manen M (1990). Researching lived experience: human science for an action sensitive pedagogy.

[55] World Health Organization (2014). Preventing suicide: a global imperative.

[56] Murthy RS, Thornicroft G, Szmukler G, Mueser KT, Drake RE (2011). Mental health services in low- and middle-income countries. Oxford textbook of community mental health.

[57] Neimeyer RA, Fortner B, Melby D (2001). Personal and professional factors and suicide intervention skills. Suicide Life Threat Behav.

[58] Spedding MF, Stein DJ, Sorsdahl K (2014). Task-shifting psychosocial interventions in public mental health: a review of the evidence in the South African context. South African Health Rev.

[59] Petersen I, Lund C, Bhana A (2012). Flisher AJ, the Mental Health and Poverty Research Programme Consortium. A task shifting approach to primary mental health care for adults in South Africa: human resource requirements and costs for rural settings. Health Policy Plan.

[60] Department of Social Development (2006). Minimum norms and standards for in-patient treatment centres.

[61] Department of Social Development (2006). Minimum norms and standards for out-patient treatment centres.

[62] Statistics South Africa (2016). Mid-year population estimates 2016.

[63] World Bank. Poverty South Africa. World Bank. 2017. http://data.worldbank.org/topic/poverty?locations=ZA. Accessed 14 Feb 2017.

[64] Iemmi V, Bantjes J, Coast E, Channer K, Leone T, McDaid D (2016). Suicide and poverty in low-income and middle-income countries: a systematic review. Lancet Psychiatry.

[65] Kalichman SC, Simbayi LC, Kagee A, Toefy Y, Jooste S, Cain D (2006). Associations of poverty, substance use, and HIV transmission risk behaviors in three South African communities. Soc Sci Med.

[66] Thompson RG, Alonzo D, Hu MC, Hasin DS (2017). Substance use disorders and poverty as prospective predictors of adult first-time suicide ideation or attempt in the united states. Community Ment Health J.

[67] Stark K, Joubert G, Struwig M, Pretorius M, Van der MN, Botha H (2010). Suicide cases investigated at the state mortuary in Bloemfontein, 2003–2007. S Afr Fam Pract.

[68] Department of Social Development (2013). National drug master plan 2013–2017.

[69] Brooke-Sumner C, Lund C, Petersen I (2016). Bridging the gap: investigating challenges and way forward for intersectoral provision of psychosocial rehabilitation in South Africa. Int J Ment Health Syst..

[70] Corrigan PW, Druss BG, Perlick DA (2014). The impact of mental illness stigma on seeking and participating in mental health care. Psychol Sci Public Interest.

[71] Reynders A, Kerkhof AJ, Molenberghs G, Van Audenhove C (2014). Attitudes and stigma in relation to help-seeking intentions for psychological problems in low and high suicide rate regions. Soc Psychiatry Psychiatr Epidemiol.

[72] Sun BQ, Zhang J (2016). Economic and sociological correlates of suicides: multilevel analysis of the time series data in the United Kingdom. J Forensic Sci.

[73] Jacob KS (2008). The prevention of suicide in India and the developing world: the need for population-based strategies. Crisis.

[74] White J, Marsh I, Kral MJ, Morris J (2016). Critical suicidology: transforming suicide research and prevention for the 21st century.

[75] Armstrong G, Samson L (2016). The imperative to integrate suicide prevention within community-based harm reduction programs for people who inject drugs: informed by the situation in Delhi, India. Int J Drug Policy.

